# Is there a role for hypnosis in palliative care?

**DOI:** 10.1177/26323524251321852

**Published:** 2025-02-25

**Authors:** Petra Vayne-Bossert, Janet Hardy

**Affiliations:** Geneva University Hospitals, Hôpital de Bellerive, 11 chemin de la Savonnière, Collonge-Bellerive 1245, Switzerland; Mater Research – University of Queensland, South Brisbane, QLD, Australia

**Keywords:** anxiety, distress, hypnosis, hypnotherapy, palliative care, quality of life, symptoms

## Abstract

Hypnosis has gained popular interest over the last few decades and has become increasingly subject to research study. Evidence of benefit in the palliative care setting is largely lacking, but it has been shown to have a favourable impact on psychological symptoms, especially anxiety, as well as chronic pain conditions. As a personalised approach, hypnotherapy is an excellent example of individual-centred care. Moreover, in the absence of significant adverse effects, it offers great promise as a complementary therapy.

## Introduction

Palliative medicine aims to relieve symptoms to enhance the quality of life of patients suffering from an advanced and incurable disease.^1^ This is one of the main differences from other medical specialties that focus on curing a disease or stabilising an organ. The palliative approach using hypnotherapy is an excellent example of patient-centred care in that the patient, guided by the therapist, plays a key role in addressing their own symptoms. There is a growing demand for integrative approaches to symptom management, most often when traditional medicine reaches its limits and patients continue to experience suffering or have unmet needs.^2,3^ Hypnosis has gained popular interest over the last few decades and has become increasingly subject to research study. Several reviews have now been published that include hypnosis as a complementary medicine approach.^4–6^ Nevertheless, evidence of benefit is still very scarce and insufficient, mainly due to small-sized trials and/or weak methodology. This practical narrative review aims to help the palliative care clinician advise the patient about the most recent advances in clinical research around hypnosis and to give guidance as to whether this approach may be useful for them.

## Methods

Pubmed/Medline and Cinhal databases were searched for MESHterms (((‘Palliative Medicine’[Mesh]) OR (‘Palliative Care’[Mesh] OR ‘Hospice and Palliative Care Nursing’[Mesh])) AND (‘Hypnosis’[Mesh] OR ‘Hypnosis, Anesthetic’[Mesh])). Furthermore, for specific symptoms such as pain, dyspnoea, nausea and anxiety individual searches for each symptom were conducted using again the MESHterms ‘Hypnosis’ OR ‘Hypnotherapy’ combined with the symptom MESHterm. The results of both searches were then analysed for relevance to our narrative review: Articles were only included if they fulfilled the following criteria: (1) included patients receiving palliative care for life-limiting disease; (2) the main intervention was hypnosis; (3) the measured outcome focused on one or several symptoms. When available, only randomised trials, systematic reviews or meta-analyses were considered otherwise other study designs were briefly described in this narrative review.

## What is hypnosis?

The NIH—National Cancer Institute defines hypnosis as ‘*A trance-like state in which a person becomes more aware and focused on certain feelings, thoughts, images, sensations, or behaviours. A person may feel calmer and more open to suggestions in order to aid in healing*’.^7^

There are many other definitions of hypnosis, most of them emphasising the presence of an altered state of consciousness or awareness in helping to modulate an unpleasant experience. Palliative care is therefore very well placed to take advantage of this technique. In general, many clinical trials on hypnosis have focused on pain as the main outcome. However, the studied pain situations are varied, ranging from chronic pain to procedure-related pain, as well as cancer pain. There are no studies where palliative care patients (with advanced and progressive disease) have specifically been addressed, although patients with advanced cancer have been included in a few trials.

During a professional hypnosis session, the patient is guided by a trained hypnotherapist into an altered state of consciousness through induction techniques. This state of mind is called a ‘trance’ which is a natural experience that an individual has several times a day.^8^ It is a state of intense focus and concentration, in which the unconscious mind is open to imaging and suggestions that are introduced by the hypnotherapist specifically tailored to the patient’s beliefs, needs and behaviour.^9^ One of the goals of hypnosis in palliative care is to have the patient experience in this altered state of consciousness, a pleasant and/or transformed experience of things that are difficult for them (e.g. symptoms, distress and anxiety) in the conscious life. The effect of this experience often continues after the session and the subconscious will integrate progressively what has been enabled during the trance. In summary, hypnosis helps to empower the patient’s self-healing capacities, giving back self-esteem and control of the situation.^10,11^

The efficacy of hypnosis is influenced by several factors. First, the patient’s belief in the intervention and their willingness to comply with therapy. Second, some studies have shown that the degree of hypnotisability also has an impact on a person’s response to hypnosis.^12,13^ Third, the context in which hypnosis takes place is also important. A personalised approach by the hypnotherapist is a fundamental necessity and an excellent example of individual-centred care. Finally, an added benefit is that very few side effects have been reported following hypnotherapy when practiced by a trained healthcare professional.^14–18^

## History

Hypnosis was particularly popular in the late 1700s when effective pharmaceutical and surgical treatment options were limited.^19^ At the beginning of the 19th century, it gained popularity in France through the founder of ‘animal magnetism’, Franz Anton Mesmer, who used hypnosis in a more dramatic and spectacular way to ‘align’ what he believed to be an invisible fluid existing in all living things.^20^

The word hypnosis in relation to the god Hypnos was introduced in the 1840s by James Braid who considered the hypnotic state to be due to muscle fatigue and sensory fatigue.^21^ In the mid-1900s, an American psychiatrist and psychologist, Milton Erickson, developed what is called today ‘modern hypnosis’ or ‘Ericksonian Hypnosis’ by mastering the language as a therapeutic tool while including patients’ expressions and perceptions in the hypnotherapy session.^22^ This type of hypnosis is now used in a wide range of conditions ranging from obesity management, insomnia, irritable bowel syndrome and functional neurological disorders.^23,24^

## Palliative care

Traditionally, symptoms of advanced disease are managed with medications, often at the cost of undesirable or unpleasant side effects. Therefore, hypnosis, an intervention with which harmful side effects are very unlikely, could be a promising complementary therapy to treat symptoms in palliative care. However, this population has so far, not specifically been subject to any randomised controlled trial (RCT) of hypnotherapy compared to other interventions or standard care. Nevertheless, chronically ill palliative care patients have been included in a 2-year-long-term follow-up study of two groups. A group provided with standard pharmacological care and early integration of self-hypnosis was compared to a group receiving standard pharmacological care alone. This cohort of 50 participants, included patients with advanced cancer, neurological or rheumatic chronic progressive diseases. Pain decreased in both groups over the 2-year period, but this was statistically more significant in the hypnosis group (*p* = 0.0001). Furthermore, the investigators observed a higher risk of increasing opioid use in the control group as well as greater anxiety than in the hypnosis group.^25^

## Pain in palliative care

Pain is a very common and bothersome symptom in palliative care and has been the subject of many studies, including hypnosis. However, all the controlled and randomised trials published so far include cancer patients only. Furthermore, pain induced either by surgery or procedures was the main focus of many trials. Women with breast cancer have been studied in four RCTs over the last 20 years.^26–29^ The overall results of these trials show a positive impact of hypnosis on pain. Other bothersome symptoms related to surgery or procedures in breast cancer patients, such as anxiety, fatigue or nausea were also favourably influenced by hypnosis. Furthermore, women receiving hypnotherapy experienced a higher level of relaxation and some of them required less propofol^27,28^ and lidocaine^28^ during anaesthesia. A cost analysis showed reduced institutional costs, mainly due to shorter surgery time in one study.^28^ Furthermore, the Society for Integrative Oncology has stated in their recently published guidelines that hypnosis can be considered an option with a moderate recommendation for procedural or surgical pain in adult cancer patients.^30^

As far as other pain situations are concerned, evidenced-based conclusions cannot be drawn. There is only one study of 68 patients treated with radiotherapy for head and neck cancers. The main objective was to analyse the efficacy of hypnotherapy on post-radiotherapy pain compared to usual care. The authors report significantly less pain in the intervention group after adjustment for gender, age and pain medication with a mean reduction in the pain score of almost 2 points on a numerical rating scale from 0 to 10.^31^

Taking the focus off cancer patients, there are no randomised control trials including palliative care patients with pathologies other than cancer. However, there is moderate evidence, published in several systematic reviews and meta-analyses, showing a positive impact on pain intensity in various chronic or acute pain situations.^32–35^ One of the recent meta-analysis including 530 participants (nine RCTs) with chronic musculoskeletal and neuropathic pain, a medium effect size was observed through a moderate decrease in pain intensity with hypnosis compared to the control interventions. It has been determined that eight sessions are necessary to achieve a moderate-to-large effect. The authors conclude, therefore, that a minimum of eight sessions are necessary to manage these chronic pain conditions.^32^

A very extensive systematic review and meta-analysis was conducted by Garland et al. in 2020 on the effectiveness of mind-body therapies for opioid-treated chronic pain. Sixty randomised control trials were included of which 23 tested hypnosis. In 65% of the studies, a statistically significant improvement in pain intensity was reported, although with a medium effect size. Twelve of these trials also showed less opioid consumption or less desire for opioids as well as a delayed time for the first postoperative opioid dose in patients receiving hypnotherapy. The study population was undergoing non-oncological surgery or procedures (eight trials), dental surgery (four trials), had severe burn pain (five trials), were trauma victims (one trial) or had a cancer diagnosis (four trials). One trial included hospital in-patients complaining of unbearable suffering with miscellaneous chronic pain disorders. There was no specific mention nor analysis of palliative care patients with non-oncological conditions.^35^

Other reviews have investigated further chronic pain conditions, such as multiple sclerosis, fibromyalgia, chronic pelvic pain and irritable bowel syndrome, and have shown inconsistent results, albeit from trials of small sample size, poor methodological quality and heterogeneous interventions.^36–39^

No conclusions can therefore be drawn on the impact of hypnosis on pain in palliative care patients, other than those with cancer undergoing surgical or other procedures. Further studies specifically aimed at pain in these patients need to be conducted.

## Dyspnoea

Dyspnoea is a very unpleasant and stressful experience of lacking air. The underlying causes are very variable, but this symptom is frequent in palliative care patients and is not restricted to those with lung disease or cancer.^40^ Dyspnoea often comes with anxiety.^40,41^ Except for one recent RCT that compared a short 15-min-individual-hypnosis session to a 15-min sham intervention in 21 COPD patients, we have been unable to find any other trial on hypnosis and dyspnoea. In this cross-over study, a 24 h washout period was allowed between the two interventions. The main outcome measure was anxiety levels, as well as respiratory signs and symptoms. The authors reported a significant decrease in anxiety and a lower respiratory rate in the hypnosis group, measured with the State and Trait Anxiety Score. A 23.8% decrease in the total score was observed in the hypnosis group versus only a 3.1% decrease in the sham group. The results were considered moderate evidence. Furthermore, both groups had improved oxygen saturation and Borg exertion scores.^42^

## Psychological symptoms

Anxiety, depression, loss of hope and even existential distress are frequent symptoms in palliative care, independent of the underlying disease. Relaxation techniques, which can also be used during a hypnotherapy session, have been shown to improve anxiety in several different situations.^43^

Cancer patients were included in the meta-analysis by Chen et al, exploring the impact of hypnosis on anxiety and psychological distress. Various types of anxiety and cancer patients were considered (including paediatric patients). The meta-analysis of the 20 trials (RCTs and pre-post-design studies) showed a significant immediate effect as well as a sustained effect on anxiety. It must be mentioned though that this effect was small and there was great heterogeneity among the studies pooled in this meta-analysis. Interestingly, higher mean effect sizes were found in European compared to American studies. Patients with procedure-related stress were particularly prone to respond to hypnosis.^44^

A meta-analysis of 17 trials using a hypnosis intervention in comparison to different control groups, such as no treatment at all, a waiting list or standard care (including standard medical or psychological attention) showed a decrease in anxiety through hypnosis with a mean weighted effect size of 0.79. Four types of anxiety were included (dental anxiety, general anxiety, test or performance anxiety and surgery or medical anxiety). None of the patients included were in a palliative care situation.^45^

Another meta-analysis of 13 RCTs evaluating the impact of hypnosis on depression and depressive symptoms, showed that the average patient experienced better symptom control with hypnosis than about 76% of the control group. Again, the study did not include palliative care patients specifically.^34^

## Nausea and vomiting

Nausea and vomiting are distressing symptoms in patients undergoing cancer treatment, but also at the end of life for various other reasons. Nevertheless, the effect of hypnosis has only been studied in cancer patients, most often in children. The most recent systematic review on hypnosis in chemotherapy-induced nausea and vomiting (CINV) was published in 2007^46^ and included six RCTs of which five were conducted in children. The authors concluded, emphasising that all the studies were small and quite heterogeneous, that hypnosis may be a valuable supplementary tool for CINV in children. To our best knowledge, there has not been further evidence on the effect of hypnosis on nausea and vomiting in children with cancer since. Nevertheless, different oncology societies (MASCC/ESMO),^47^ as well as the recently updated Clinical Practice Guidelines^48^ on the prevention and treatment of anticipatory chemotherapy-induced nausea and vomiting in paediatric cancer patients and haematopoietic stem cell recipients indicate—based on low-quality evidence—that hypnosis can be offered as secondary prevention of CINV especially in children with cancer.

In one study with the adult population, 67 patients undergoing bone marrow transplantation were randomised to hypnosis, cognitive coping, therapist attention or usual care.^49^ There was no statistically significant difference in nausea or vomiting between the different groups. In one further prospective controlled study, women diagnosed with ovarian cancer were invited to join a trial of a combined intervention, including hypnosis, therapeutic massage and healing touch during chemotherapy versus chemotherapy alone. One of the outcomes was the use of anti-emetic medication; no difference was found between the intervention and standard care groups (5.95 prescriptions on average in the integrative medicine arm and 4.75 prescriptions in the control arm, *p* = 0.09).^50^

## Safety and satisfaction of hypnosis

Very few studies focus on the potential side effects of hypnosis. A global review of adverse events documented in clinical studies of hypnosis reported no serious adverse event and a very low incidence of all other side effects (<1%), of which dizziness, a sensation of floating or heaviness and headaches were the most common.^51^ It must be acknowledged, however, that there are no standardised methods to assess and report adverse events of hypnosis and very few trials even report such events. Patients may report some unusual sensations or feelings, experienced as part of the dissociation process during the trance. An experienced hypnotherapist uses such responses to direct the therapeutic process. Sometimes patients experience a prolonged sensation of lowered alertness after the session. A well-trained hypnotherapist can handle these reactions properly and provide a safe environment for the patient.^52,53^

Several trials have reported patient or health care professional’s satisfaction during trials of hypnosis.^15,54,55^ Both parties have reported experiencing an enhanced feeling of relaxation and calm especially regarding procedures or interventions.^56^

Even in the event of little or no impact of hypnosis on the targeted symptom, general satisfaction with this intervention has been reported by patients. Hypnosis enhances the patient’s perception of control over pain and increases a sense of relaxation and well-being due to a decrease in perceived stress.^57^ In an oncology trial, some patients even reported a more personal level of connection with the nurse during chemotherapy treatment.^58^

## Discussion

Hypnotherapy is an approach that has been used for centuries, initially exclusively in psychiatry. In the last decades, it has gained interest in the fields of dentistry and anaesthesiology with a focus on pain control during surgical interventions. In the context of palliative care where symptom control is the main focus, hypnotherapy by also enhancing patient empowerment and self-control, is being offered more frequently to patients with an advanced cancer disease. However, many of the studies published in the literature are small and uncontrolled, with no standard outcome measures. The few RCTs published in the literature show not only a positive impact of hypnotherapy on the targeted symptom, but generally a positive impact on the overall well-being of the participants. An advantage of hypnotherapy is, that if practiced by a trained healthcare professional, it has a very favourable benefit-to-harm ratio and is, therefore, an excellent example of patient-centred therapy.

The main reason why research with hypnotherapy remains challenging is that every session is tailored to the patient’s needs and expectations. The latter is especially important, since patients with a positive mindset to hypnosis or complementary approaches in general, experience better outcomes with these therapies.^13^ Therefore, standardised hypnotherapy sessions may be less effective.^59–61^

## Conclusion

Even in the absence of clear evidence of benefit in palliative care patients, hypnotherapy may help these patients in coping with various symptoms, regardless of the stage of their disease. More methodologically sound studies with bigger sample sizes are needed. Perhaps in the future, hypnosis will be considered standard care for any patient in need of it. Because of the paucity of research in this area and the consequent lack of evidence of benefit in many areas, hypnosis can currently only be recommended for pain control in women with breast cancer undergoing surgical procedures and for secondary prevention of chemotherapy-induced nausea/vomiting in children. Although not formally studied in the palliative care population, hypnosis can have a favourable impact on psychological symptoms, especially anxiety, as well as chronic pain conditions, including the lower use of opioids.

Quote from Milton Erickson: ‘You use hypnosis not as a cure but as a means of establishing a favourable climate in which to learn.’

## Declarations

**Ethics approval and consent to participate:** Not applicable.

**Consent for publication:** Not applicable.

**Availability of data and materials:** Not applicable.
